# Restless Legs Syndrome among Pakistani Population: A Cross-Sectional Study

**DOI:** 10.1155/2015/762045

**Published:** 2015-01-27

**Authors:** Khalid Mahmood, Rabeea Farhan, Asif Surani, Arif Anwer Surani, Salim Surani

**Affiliations:** ^1^Dow Medical College and Civil Hospital Karachi, Sindh 74200, Pakistan; ^2^Dow University of Health Sciences, Karachi, Sindh 74200, Pakistan; ^3^Texas A&M University, College Station, TX 77843, USA

## Abstract

*Objective*. Restless leg syndrome (RLS) is a chronic distressing disease characterized by an urge to move the legs with an unpleasantsensation during periods of rest. The global prevalence estimates of RLS range from 2.5% to 15%.* Method*. This cross-sectional study was conducted at various hospitals in Karachi during August 13 to March 14. The visitors who had accompanied patients to Outpatient Department or had come to visit admitted patients were approached conveniently. Subjects were interviewed regarding the essential criteria of RLS and its attributes.* Results*. The sample size was 390 with 56% being females. The point prevalence of RLS was estimated to be 23.6%. The prevalence in females was twice as high as compared to males. Smoking and low level of education were associated with RLS (*P* < 0.001). Among RLS positive individuals, 50.1% reported frequency of their symptoms to be greater than 16 days per month and 64.1% graded their symptom severity as mild to moderate. About 37% of RLS positive individuals consulted a general physician for their symptoms.* Conclusion*. RLS is highly prevalent and underdiagnosed condition among Pakistani population. Efforts must be directed to raise the awareness of this condition among physicians and general population.

## 1. Introduction

Restless leg syndrome (RLS) is a chronic distressing condition. It is characterized by a discomforting sensation of the limbs with an urge to move legs at rest and inactivity. This urge is often accompanied by uncomfortable sensations like tingling, creeping, soda bubbling, or burning [1]. For improved recognition and diagnosis of this condition, the international restless leg syndrome study group (IRLSSG) has published the clinical diagnostic criteria for RLS [1]. The four essential criteria which must be met for the positive diagnosis include an urge to move legs accompanied by unpleasant sensation, beginning during periods of rest and inactivity, worsened in evening or at night, and partially or totally relieved by movements. Response to dopaminergic treatment, periodic limb movement, and positive family history were designated as supportive criteria for the diagnosis. RLS can be classified as a primary disorder having idiopathic origin or occurring secondary to pregnancy, iron deficiency, renal failure, polyneuropathies, diabetes mellitus, and anemia [2–6].

Recent studies have reported significant prevalence of RLS among general population. Although variations exist in the criteria used to classify RLS, the researches report prevalence of RLS among general population to be 2.5–15% [6–8]. Among western population the prevalence was estimated to be comparatively higher than Asian population [9–12]. In ethnically comparable population, India, the RLS prevalence was found out to be 2.1% [13]. Despite the highly reported prevalence, the incidence of RLS is still assumed to be underestimated. The unawareness of general practitioners and misdiagnosis of RLS as anxiety, depression, and varicose veins makes this disorder even more concealed within general population [14].

Various associations and risk factors of RLS have been delineated by the literature. Numerous studies have indicated the prevalence of RLS to be twice as high in women compared to men [8]. The literature has also specified that most cases of idiopathic RLS have an age of onset at 33–36 years [8]. However, the association with increasing age is not conclusive. The literature has also established that individuals with obesity, diabetes, and hypertension have a greater likelihood of having RLS [8]. Other documented risk factors shown in literature are family history, smoking, alcohol, and use of SSRI [8, 15].

RLS poses a significant impact on health related to quality of life and a considerable economic burden comparable to a chronic disorder [16, 17]. RLS has been positively associated with insomnia, impaired sleep quality, and excessive daytime sleepiness [8]. To date, there is no published data reporting prevalence and association of RLS among Pakistani general population. This pilot study intends to estimate the prevalence of RLS in the general population of Pakistan and evaluate its associated risk factors.

## 2. Methods

After approval from ERB this cross-sectional study was conducted during August 2013 to March 2014. The study setting comprised of two government sector and two private sector hospitals. These hospitals have a busy outpatient and inpatient departments and cater a diverse population from all walks of life. Individuals from different part of country visit these major hospitals for consultation regarding their health issues. As a cultural norm, attendants, usually relative or a friend, accompany the patient in Outpatient Department (OPD) or visit admitted patients during visiting hours. These attendants were focused in this study and the data was sampled from them.

The sample size of the study, assuming 50% prevalence, keeping confidence level at 95% and accepting 5% margin of error, was estimated to be 385. The required sample size was rounded off to 390 subjects. The interview was conducted in the national language Urdu. The data collection team comprised of final year medical students who were in-serviced and trained about the RLS and administering the questionnaire. Individuals aged 18 or above were included, while individuals who are suffering from stroke or have a history of limb weakness or impaired sensation, history of trauma to the limbs, and active diseases other than chronic conditions like hypertension, diabetes, kidney, or heart disease were excluded. After evaluating the patient's eligibility to participate in the study, an informed written consent was obtained. Study questionnaire was then administered to the subjects.

The study questionnaire was divided into three parts. The first part of the questionnaire gathered demographic information including age, gender, height and weight, marital status, educational background, chronic medical conditions, and smoking status of the subject. The second part inquired about the four essential criteria of RLS specified by IRLSSG. The questions included were

1. Are you facing any discomfort or unpleasant sensation in your legs like tingling, crawling with ants, or pain accompanied with an urge to move legs?
2. Does this unpleasant sensation or urge to move legs begin or worsen during periods of rest or inactivity?
3. Does this unpleasant sensation or urge to move legs partially or fully relieve by movements such as walking, stretching, or wiggling feet and toes?
4. Does this unpleasant sensation or urge to move legs worsen in the evening or at night compared to during the day or only occur during the evening or night?

Individuals meeting all four essential criteria were considered positive for RLS; and the third part of questionnaire was only administered to these RLS positive individuals. In the third part, the severity and associated features of RLS symptoms was explored. It commences with the assessment of frequency and severity of such symptoms and is followed by questions regarding medical consultation for these symptoms. The aggravating and relieving factors, impact on sleep quality, and occurrence of repeated jerking movement during sleep (periodic limb movement) were also sought. Lastly, the presence of such symptoms in first degree family relatives was inquired upon.

The data obtained was double entered and was analyzed in Statistical Package for Social Sciences 17.0 (SPSS, Inc., Chicago, IL, USA). The qualitative variables were expressed as percentages, and quantitative variables were expressed as mean ± standard deviation. A *P* value < 0.05 was regarded as statistically significant.

## 3. Results

The data of 390 subjects was included in the final analysis. Overall there were 218 (55.9%) females and 172 (44.1%) males. The mean age was 36.5 ± 14.64 and mean BMI was 24.0 ± 4.49. Other characteristics of sample population are summarized in Table 1.

Out of 390 individuals, 92 (23.6%) were classified as RLS positive based on criteria defined by IRLSSG. The prevalence of RLS was 66 (30.3%) and 26 (15.1%) among females and males, respectively, with a *P* value < 0.001. The prevalence of RLS positive subjects was found to increase with increasing age till age group of 31–40 which showed a peak prevalence of RLS. Thereafter, there was no significant difference in prevalence among the older age groups. However, the prevalence still remains at a higher level in older age group compared to individuals age <30 years (*P* value = 0.001). There was no statistically significant relation found between BMI and prevalence of RLS positive subjects.

The prevalence of RLS was also noted to be statistically significant among smokers (*P* value = 0.001). However no correlation was found between numbers of cigarette smoked per day and prevalence of RLS. Social factors like low level of education were also associated with increased prevalence of RLS positive individuals (*P* value ≤ 0.001). In addition, RLS prevalence was also noted to be high among individuals with multiple comorbidities (*P* value = 0.001).  Table 2 further elaborates different characteristics of sample population by individuals RLS status.

Among RLS positive subjects, the reported frequency of RLS symptoms was greater than 16 days per month in 50.1% individuals. There was a statistically significant relation between increased frequency of RLS symptoms and female gender (*P* value < 0.05). Regarding severity of such symptoms, 64.1% RLS positive subjects have rated their symptoms mild to moderate. Only 10.9% subjects have rated their symptoms to be very severe. Despite the increased reported frequencies of attack, only 37% individuals have ever consulted a general physician for their symptoms. The presence of self-reported periodic limb movement was indicated by 33.7% subjects while the presence of RLS symptoms in close family members was given by 53.3% individuals. There were no objective tests done to confirm the presence or absence of periodic limb movement in the subjects.

Stress was observed to be the most frequent aggravating factor while gentle massage was reported to be a common relieving factor. Concerning the impact of RLS symptoms on sleep quality, only 6.3% subjects have regarded their sleep quality to be poor.  Table 3 elaborates various characteristics of symptoms experienced by RLS positive individuals and also shows genderwise distribution.

## 4. Discussion

This was the first attempt to characterize the prevalence of RLS and its relationship with various factors among general population in Pakistan. This study has shown a point prevalence of 23.6% with female preponderance. RLS was found to be positively associated with smoking and low level of education. It was observed that RLS symptoms appeared at a much higher frequency and were rated as mild or moderate on the severity scale. It was striking to note that almost two-thirds of RLS positive subjects have never consulted a physician regarding their symptoms.

The reported point prevalence of 23.6% in our study is considerably higher than the prevalence of RLS reported elsewhere. Studies conducted in Asian countries yielded a point prevalence ranging from 1.1 to 2.1% [12, 13, 18], while those from western countries showed a higher estimate of prevalence ranging from 4 to 29% [19]. All these studies employed the same IRLSSG essential criteria as used in our study. Thus, the reported point prevalence of 23.6% in our study is comparable to the prevalence estimates in western population. The difference between reported RLS prevalence by our studies and studies conducted elsewhere in Asia can be partly explained by scarcity of data from this region of the world, leading to underestimation of true prevalence. Besides, less rigid sampling methods employed in our study can also be responsible for such significant difference in prevalence.

The finding of twice as high prevalence of RLS in female gender compared to men in our study relates to the findings concluded by other studies [11, 15, 18, 20]. Furthermore, the finding of peak prevalence of RLS in age group of 31–40 years is also consistent with findings of Ohayon and Roth [15] and Bjorvatn et al. [20]. The reason behind presence of such findings in our study is unclear. It can possibly be due to higher incidence of iron deficiency anemia and low ferritin level which is highly prevalent among females in the developing countries; however, Bjorvatn and colleagues attributed this finding to the fact that older individuals can have prolonged duration of RLS symptoms. Thus they can inappropriately respond “No” to the fourth essential criteria of RLS which inquires about worsening of RLS symptoms only at evening or night. This can lead to false negative labeling of such subjects and underestimation of prevalence in older subjects [20].

Various social factors have been found to influence the prevalence of RLS. The relationship between cigarette smoking and RLS symptoms had been explored by various studies and was determined to be equivocal. A cross-sectional study conducted by Ohayon and Roth showed an increased prevalence of RLS symptoms among individuals who have smoked more than 20 cigarettes per day [15]. However, Lavigne and colleagues have regarded this relation as statistically insignificant [21]. The finding of increased RLS prevalence among smokers in our studies is comparable to the finding of Ohayon and Roth. A correlation between number of cigarettes smoked and presence of RLS symptoms could not be inferred from our data. Our study has also highlighted a potential impact of low level of education on RLS prevalence. This finding is similar to the findings of study conducted by Rangarajan and colleagues among Indian population [13]. This reported association in our study can be explained by the fact that individuals with low level of education are usually at poor socioeconomic status, making them more susceptible to poor nutrition including iron deficiency and parasitic infestation. These factors could serve as a secondary cause for RLS and can account for increasingly reported frequency in this specific subset.

Numerous studies conducted across the world have shown the frequency of RLS symptoms to be twice per week in majority of subjects. In literature it has also been observed that most RLS positive individuals regard the severity of their symptoms to be moderate to severe [8]. In our study, the reported frequency of symptoms in RLS positive individuals was greater than 16 times per month (four times per week) in almost half of our subjects. This increased reported frequency can be due to erroneous interpretation of such symptoms or confusing symptoms of tiredness or fatigue with symptoms of RLS. In addition, our study has not shown a statistically significant relation between impaired sleep quality and RLS symptoms. This finding differs from the heavily published data regarding adverse effect of RLS on sleep quality. This discrepancy can be attributed to the low sample size and the lack of structured questionnaires to evaluate sleep quality among subjects in this study.

A striking observation made from our study was the lack of physicians' consultation for the symptoms of RLS among RLS positive individuals. In an attempt to identify factors behind such behavior, Bjorvatn and colleagues have concluded that most patients believe that their symptoms are not severe enough to involve a doctor, while a small percentage felt that doctors could not help them with their problem [20]. Likely explanation in our population can be lack of perception of RLS as a disease entity and erroneously assuming that these symptoms have originated as a result of exertion or fatigue.

Our study has some limitations. Firstly, the convenience sampling method employed in this study and the selection of visitors as sample subjects restrict the generalization of our results to the entire population. Second, no measure was taken to exclude the RLS mimics. In addition, the screening of subjects for exclusion criteria and presence or absence of various medical conditions was based on subjective responses. There were no objective tests done to confirm the presence or absence of these findings which can limit the strength of our analysis. Also, the various ethnic backgrounds and cultural disparities were not taken into account due to their diversity, cross ethnic marriages and challenge to identify various ethnic subtypes. Furthermore, the questionnaire used was translated by the interviewer and the translation was not validated. Nevertheless, the use of standardized criteria recommended by IRLSSG enhances the validity of our findings. Further studies are suggested to explore this important issue in this population.

## 5. Conclusion

In conclusion, the prevalence of RLS is determined to be significant among Pakistani population. Further studies with randomized sampling employing an objective approach are required to better characterize the prevalence and associations of RLS. Moreover, extensive local and regional efforts are required to raise the awareness of RLS among general population and physicians.

## Figures and Tables

**Table 1:** Characteristics of sample population with mean (± standard deviation) and percentages.

Characteristics	Mean ± SD and percentages
Marital status	
Single	37.2%
Married	61.3%
Divorced	1.5%
Education	
No formal education	12.3%
Below Grade 10	6.9%
Grade 10	6.9%
High school	8.2%
Undergraduate	14.6%
Graduate	51.0%
Smoking	
Current smokers	7.4%
Cigarettes smoked/day	6.64 ± 2.3
Comorbid conditions	
Ischemic heart diseases	1.5%
Diabetes	2.6%
Hypertension	8.7%
Multiple	7.9%

**Table 2:** Characteristics of sample population by RLS status.

Characteristic	RLS status		*P* value
	Positive *n* (%)	Negative *n* (%)	
Gender			
Female	66 (30.3)	152 (69.7)	<0.001
Male	26 (15.1)	146 (84.9)	
Age			
<20	3 (12.0)	22 (88.0)	0.001
21–30	22 (13.7)	139 (86.3)	
31–40	27 (38.0)	44 (62.0)	
41–50	15 (29.4)	36 (70.6)	
51–60	18 (31.6)	39 (68.4)	
61–70	7 (31.8)	15 (68.2)	
>70	0	3 (100)	
BMI			
Underweight	5 (14.3)	30 (85.7)	0.072
Normal (18–25 kg)	45 (21.0)	169 (79.0)	
Overweight	26 (26.8)	71 (73.2)	
Obese (>30 kg)	16 (36.4)	28 (63.8)	
Marital status			
Married	50 (20.9)	189 (79.1)	0.10
Unmarried	18 (12.4)	127 (87.6)	
Divorced	2 (33.3)	4 (66.7)	
Educational level			
No formal education	22 (45.8)	26 (54.2)	<0.001
Below Grade 10	11 (40.7)	16 (59.3)	
Grade 10	10 (37.0)	17 (63.0)	
Intermediate	5 (15.6)	27 (84.4)	
Undergraduate	8 (14.0)	49 (86.0)	
Graduate	36 (18.1)	163 (81.9)	
Smoking			
Yes	14 (48.3)	15 (51.7)	0.001
No	78 (21.6)	283 (78.4)	

**Table 3:** Characteristics of symptoms of RLS positive individuals.

Characteristics	Female *n* (%)	Male *n* (%)	Total *n* (%)
Frequency of attacks^*^			
>16 times per month	40 (60.6%)	06 (23.1%)	46 (50.1%)
5–15 times	09 (13.6%)	06 (23.1%)	15 (16.3%)
2–4 times	10 (15.2%)	05 (19.2%)	15 (16.3%)
Rarely or <1 time	07 (10.6%)	09 (34.6%)	16 (17.3%)
Severity of attacks			
Mild	20 (30.3%)	10 (38.5%)	30 (32.6%)
Moderate	20 (30.3%)	09 (34.6%)	29 (31.5%)
Severe	19 (28.3%)	04 (15.4%)	23 (25.0%)
Very severe	07 (10.9%)	03 (11.5%)	10 (10.9%)
Consultation			
Yes	26 (39.4%)	08 (30.8%)	34 (37%)
No	40 (60.6%)	18 (69.2%)	58 (63%)
Sleep quality			
V. good	08 (12.1%)	05 (19.2%)	13 (14.1%)
Fairly good	34 (51.5%)	13 (50.0%)	47 (51.1%)
Fairly bad	19 (28.8%)	07 (26.9%)	26 (28.3%)
Very bad	05 (07.6%)	01 (03.8%)	06 (06.5%)
Periodic limb movement			
Yes	21 (31.8%)	10 (38.5%)	31 (33.7%)
No	45 (68.2%)	16 (61.5%)	61 (66.3%)
Family history			
Yes	34 (51.5%)	15 (57.7%)	49 (53.3%)
No	32 (48.5%)	11 (42.3%)	43 (46.7%)

^*^ *P* value < 0.05.
